# Patient experiences align with the familial hypercholesterolemia global call to action

**DOI:** 10.1016/j.ajpc.2022.100344

**Published:** 2022-04-18

**Authors:** Laney K. Jones, Nicole Walters, Andrew Brangan, Catherine D. Ahmed, Katherine A. Wilemon, Gemme Campbell-Salome, Alanna K. Rahm, Samuel S. Gidding, Amy C. Sturm

**Affiliations:** a Genomic Medicine Institute, Geisinger, Danville, PA, United States of America; b Heart and Vascular Institute, Geisinger, Danville, PA, United States of America; c Family Heart Foundation, Pasadena, CA, United States of America

**Keywords:** Familial hypercholesterolemia, ASCVD, Prevention, ASCVD, atherosclerotic cardiovascular disease, FH, familial hypercholesterolemia, LDL-C, low-density lipoprotein cholesterol

## Abstract

**Objective:**

To explore alignment of perspectives from individuals and families with familial hypercholesterolemia (FH) to the FH Global Call to Action recommendations.

**Methods:**

Interviews and focus groups were conducted with individuals and families with FH from multiple U.S. health systems and the Family Heart Foundation community to capture lived experiences and to identify barriers to diagnosis, cascade testing, and treatment. Participant perspectives were examined and classified, according to their alignment to recommendations of the FH Global Call to Action.

**Results:**

A total of 75 lived experiences were analyzed. Participants were majority female, mostly white, older, and well-educated. Participants most frequently mentioned recommendations were family-based care (84%) and screening, testing, & diagnosis (84%), followed by treatment (69%), advocacy (60%), cost & value (59%), awareness (56%), research & registries (43%), and severe & homozygous FH (11%). An average of 4.65 (SD 1.76) recommendations were mentioned.

**Conclusions:**

The FH Global Call to Action was driven by the persistent unmet needs of those living with FH in receiving a timely diagnosis, appropriate care, and support to prevent early morbidity and mortality. Patient- and family-centric perspectives suggest the FH Global Call to Action captures these concerns. Acting on recommendations, particularly improvements in screening and family-based care, will address patient, and public health, concerns.

## Graphical abstract

**Figure:**
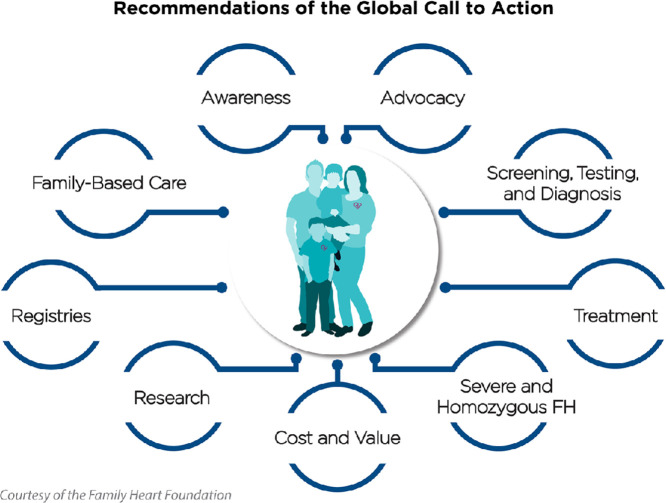

## 1 Introduction

Familial hypercholesterolemia (FH) is a genetic disorder that leads to premature morbidity and mortality due to atherosclerotic cardiovascular disease (ASCVD)[1,2]. Approximately 10% of individuals with FH are diagnosed and this diagnosis often comes late; furthermore, once diagnosed, many receive suboptimal treatment[2]. This creates a major public health problem as FH is relatively common (frequency of 1:250) yet there is low awareness of the condition and most people with the condition are undiagnosed[2].

**Fig. 1:**
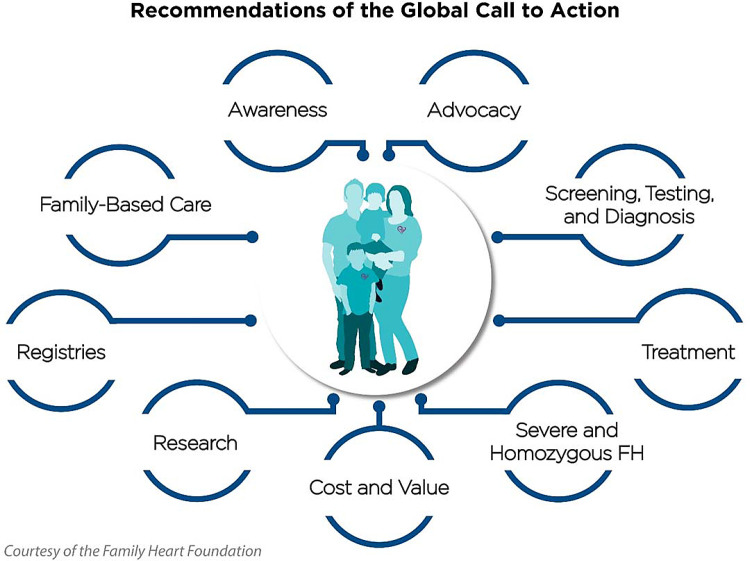
FH Global Call to Action Recommendations.

The 2020 FH Global Call to Action, led by the Family Heart Foundation (formerly the FH Foundation) and the World Heart Federation that included over 40 FH advocacy organizations worldwide, individuals with FH, and scientific experts, provides recommendations to reduce premature death and disease and the public health burden of FH[3]. The nine recommendations are based on the original recommendations accepted by the World Health Organizations’ 1998 report [4] recognizing FH as a public health priority. The nine recommendations define areas of engagement needed to educate about the condition, identify affected individuals and families, improve care, and optimize scientific advances in treatment. The nine recommendations cover: 1) advocacy, 2) awareness, 3) screening, testing, & diagnosis, 4) treatment, 5) severe & homozygous FH, 6) family-based care, 7) registries, 8) research, and 9) cost & value (Fig. 1).

How the lived experience from individuals and families with FH align with and embody the priorities outlined in the Global Call to Action could inform public health efforts to improve FH awareness, diagnosis, and management of this preventable disease. As part of ongoing research on improving FH diagnosis and care, interviews and focus groups were conducted with individuals and families with FH. Lived experiences recorded in this research, like the one below, were mapped to the nine recommendations to assess alignment. “My provider knew that my father died at 37 from a heart attack and I had elevated cholesterol and I heard, ‘Oh, you don't need to start statins because you are still premenopausal, so you don't need to worry, you have protection of estrogen’” (Participant 36).

## 2 Methods

Institutional review board approved interviews and focus groups were conducted, as a part of funded research studies[5, 6], with individuals and families with FH from multiple U.S. health systems (Geisinger, Mercy Health System, Barnes Jewish Hospital) and the Family Heart Foundation community (FH volunteer advocates from all over the country were convened for an advocacy training in Arlington, VA) to identify barriers to diagnosis, cascade testing, and treatment (Table 1)[5,6]. Patient-centered perspectives were sought in these studies to develop solutions that can be implemented to improve FH care[7,8].

**Table 1:** Data source and participants.

**Data source**	**Study**	**Participants**	**Purpose of study**	**Sample representation**	**Invited**	**Participated**
Interviews	3	Dyads (Individuals with FH and 1 family member)	Feedback on family sharing and cascade testing tools	Family Heart Foundation Advocates, Healthcare system	69	11 dyads, 22 individuals
	2*	Individuals with FH	Experiences related to FH management	Healthcare systems	189	33
Focus groups	1	Individuals with FH	Acceptability, Appropriates, Feasibility of identification and cascade screening methods	Family Heart Foundation Advocates, Healthcare system	84	22
	2*	Individuals with FH	Member checking with participants from study 2 interviews and brainstorming potential solutions	Healthcare systems	189	14
					**Total**	**75**⁎⁎

⁎ Study 2 conducted interviews with 33 participants who subsequently 14 of them participated in focus groups.

⁎⁎ A total of 2 individuals had participated in multiple focus groups or interviews between studies 1, 2, and 3.

Participant perspectives were examined and classified according to their alignment to recommendations within the Global Call to Action[3]. Study specific definitions were developed for the recommendations (Table 2). Three study team members independently coded each transcript to the nine recommendations. For this work, registries were included in the research category as participants did not distinguish between the two, leaving eight categories for classification. Two recommendations, screening, testing, & diagnosis, and treatment, were coded at the individual-level if they directly related to the individual. The other six were coded at the family-level if they referred to implications for the individual and/or their family members. Discrepancies were resolved by consensus with the coding team. Atlas.ti software version 8.4.15.0 was used to facilitate analysis and compare themes across recommendations. Descriptive statistics were calculated in Microsoft Excel.

**Table 2:** Study specific definition of the recommendations from the FH Global Call to Action study specific definitions.

**Recommendations**	**Study-specific definition**
Awareness	• Perspectives on current awareness of FH • Importance of improving awareness of FH
Advocacy	• Mentions of patients advocating for FH for themselves or their family • Mentions of clinicians advocating for their patients • Mentions role of advocacy groups
Screening, Testing, and Diagnosis	• Mentions of who, when, and how the individual was screened, tested, or diagnosed with FH • Mention of need for more screening • Mention of benefit or harm of screening
Treatment	• Mentions of medications that patients have been prescribed, any side effects, or concerns regarding the medications • Mention of diet change
Severe and Homozygous FH (HoFH)	• Definition of severe/HoFH: mentions of HoFH or very high low-density lipoprotein cholesterol (LDL-C) levels • Stories mentioning the care severe/HoFH patients received • Need for unapproved treatments, apheresis, liver transplantation
Family-based Care	• Mentions of family sharing • Discussion of family culture related to healthcare • Mentions of cascade screening of family members • Involvement of family unit in FH care
Registries	• Mentions of the utility of registry data • Participates in a registry
Research	• Individuals with FH stating FH data in relation to their story (e.g., knowledge of the data) • Suggestions on future research that should be conducted that would help improve care they have received • Individuals encouraged to participate in research to help further the field • Mention of FH mechanism
Cost and Value	• Financial cost to an individual due to having a diagnosis of FH (e.g., medical care, treatment, procedures, testing, health insurance coverage) • Impact of having a diagnosis of FH on an individual's life (in relation to health insurance, life insurance, medications) • Value of early diagnosis, prevention to improve care of those with FH

## 3 Results

**Fig. 2:**
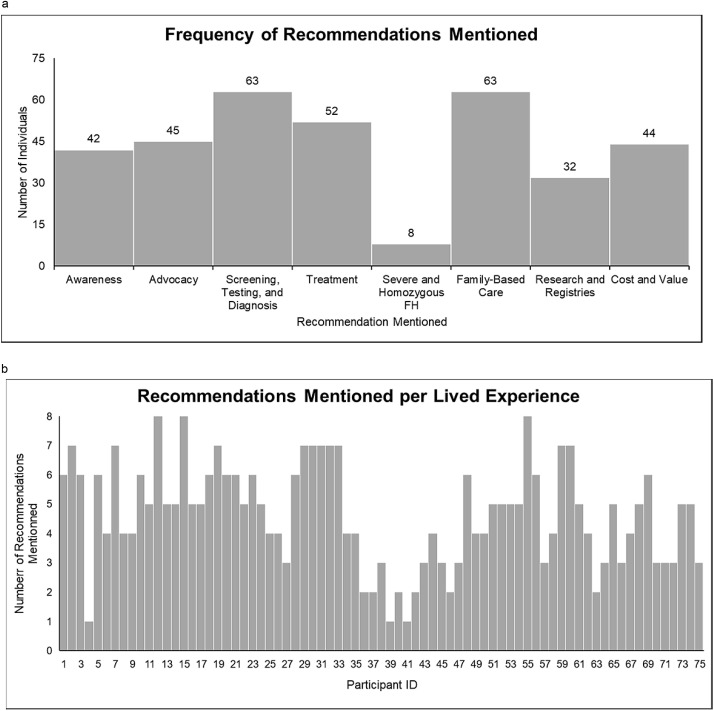
a. Histogram of recommendations mentioned by participants Fig. 2b. Histogram of 75 participants and the number of recommendations mentioned in their lived experience.

A total of 75 lived experiences from interviews and focus group transcripts were analyzed. There were two individuals who participated in more than one interview or focus group across different studies and one study conducted both interviews and focus groups with the same individuals (Table 1 and 3). Participants were majority female (58%), white (96%), older (71% were 45 years of age or older), and well-educated (60% were at least college graduates) (Table 3). The most frequently mentioned recommendations from participants’ experiences were family-based care (84%) and screening, testing, & diagnosis (84%), followed by treatment (69%), advocacy (60%), cost & value (59%), awareness (56%), research & registries (43%), and severe & homozygous FH (11%) (Fig. 2a). An average of 4.65 (SD 1.76) recommendations were mentioned by each participant (Fig. 2b). Note that many perspectives overlap across several recommendations, particularly family-based care.

**Table 3:** Demographics of patients and family members participants from interviews and focus groups.

**Demographics (*n* = 75)**	**Value**
Female, n (%)	58 (77)
Hispanic or Latino Origin, yes, n (%)	2 (3)
Race, n (%)	
White	72 (96)
Black	2 (3)
Other	1 (1)
Age Range, years, n (%)	
18 to 24	3 (4)
25 to 34	10 (13)
35 to 44	9 (12)
45 to 54	12 (16)
55 to 64	18 (24)
65 or older	23 (31)
Highest Education Level, n (%)	
Some High School	2 (3)
High School Graduate	8 (11)
Trade, Technical, or Vocational School	2 (3)
Some College	18 (24)
College Graduate	27 (36)
Post-Graduate Work or Degree	18 (24)
Annual Household Income, USD, n (%)	
Declined to Answer	12 (16)
<15,000	4 (5)
15,001–30,000	7 (9)
30,001–50,000	5 (7)
50,001–75,000	13 (17)
75,001–100,000	11 (15)
100,001–150,000	14 (19)
150,001–200,000	6 (8)
>200,000	3 (4)
Currently Married or Living with Partner, yes, n (%)	60 (80)
Currently Working for Pay, yes, n (%)	43* (58)
Health Insurance Status, yes, n (%)	74 (99)

⁎ 1 Individual Declined to Answer.

### 3.1 Family-based care

Participants living with FH raised several aspects of family-centered care, beginning with the fact that FH affects many people in one family. Most participants mentioned sharing their FH results with family members; however, the experience and uptake of cascade testing to diagnose additional family members varied. Some participants found family members receptive to this information, while others found that family members avoided or reacted negatively to the information. Family members receptive to learning about their risk for inheriting FH often underwent screening for FH. Participants discussed the benefits of sharing this information with family members, especially children and grandchildren, as it enables them to receive earlier care.

“I also know when I first found out through my lipid doctor that this was going on with me, and I reached out to my siblings, it's overwhelming for the person finding out that they have it. So, some of the questions that they had, I couldn't even really give them answers to, even though I talked to the doctor, and taken notes.” (Participant 55)

Another theme that was consistently elevated by individuals interfacing with the health system was the potential merit of family appointments, which would allow the immediate and extended family to learn about FH from a medical professional and be screened. This innovation would allow for care planning and for the entire family unit to make important decisions regarding care of their loved ones.

“We're supporting it now through generations of it, and I think it is important for those relatives, especially kids need it, to invite them to a doctor appointment with you so they could hear from a specialist and have the right information.” (Participant 45)

Other participants discuss how health-related problems were seldomly discussed within their family units; so, family appointments would not be an option that their family members would entertain.

### 3.2 Screening, testing, & diagnosis

Participants emphasized the need for and importance of early screening, testing, and effective communication of the diagnosis to prevent future ASCVD. Most participants expressed knowing about either a personal or family history of high cholesterol long before their FH diagnosis. Some participants expressed that this history or having a cardiac event led to their FH diagnosis, while others were surprised to learn about their FH diagnosis while being evaluated for another health concern. Many stated they had not been given the name for their diagnosis, ‘Familial Hypercholesterolemia’, until recently. The diagnostic journey (i.e., experience from first seeking medical care for relevant symptoms to receiving the correct diagnosis) of participants also varied, with some participants diagnosed via lipid or genetic testing, and others via population-based genomic screening.

“I found out about 4 months ago that I have FH through a genetic test. I pretty much figured that I did have that because my sister died at 38 years old from a heart…heart attack, massive. … and my niece has had 5 open heart surgeries already, and she is just now 36 years old. Her daughter is on statins, and she is only 7. … my daughter is on statins, she's 20.” (Participant 48)

## 3.3 Treatment

FH diagnosis being made later in life has led to delayed treatment. Participants reported that having the FH diagnosis made a difference in the treatment of their high cholesterol, specifically mentioning their own and their clinician's willingness to begin treatment, consideration of additional therapies beyond statins, and understanding of cholesterol goals. Some mentioned that when they were younger not all of the current treatment options were available, and others did not want to take any medications at all before they understood their diagnosis. Some shared that they are not able to achieve cholesterol levels their clinicians recommend, despite following prescribed regimens. There was also a desire by participants to have more options for medication therapy, especially ones with fewer side effects.

“I just wish I would be able to get the treatment earlier because I worry. Like I said, I don't worry about it as much now … since I started [a PCSK9 inhibitor]. But before that I would always worry like I have [to] exercise, I have to eat good … So, I guess earlier entrance into the treatment realm would probably be a good thing.” (Participant 21)

### 3.4 Advocacy

Participants spoke about advocacy on several levels: one-on-one with family members and other individuals, benefiting from the advocacy of clinicians on their behalf, and advocating on a policy level for improved diagnosis and care for FH families. Once diagnosed, many participants educated family members about the importance of FH and the need to be screened. Participants described their approaches to communicating with family members via text message, social media, and with printed material, among other methods. Participants shared their appreciation of their clinicians advocating on their behalf with health insurance companies for coverage of testing and treatments. Participants mentioned organizations such as the Family Heart Foundation that provide resources to help them advocate for their own care and that advocate on a policy level to address FH as a public health priority.

“I'm not going to give up, um, and I'm going to keep on saying it and the more stuff I learn and the more pamphlets I get and the more materials I have, I'm going to keep on telling them because I–I know they got it.” (Participant 32)

“The poor woman who's fighting for me [the] nurse practitioner and she's fighting for me all the time and I seem to take up more time, her time than I should just trying to get [treatment approved].” (Participant 19)

### 3.5 Cost & value

Participant concerns about the cost of managing their FH and resultant ASCVD and its impact of their quality of life was a significant and widespread topic. Some participants discussed the inability to afford medical care or treatment for a variety of reasons, including the high cost of testing (genetic testing specifically), medications, and lack of health insurance. Others discussed the time and effort it takes themselves and their clinicians to navigate insurance coverage and the resulting delays in treatment. Participants discussed the significant value in early identification and treatment of FH to prevent the premature ASCVD that results in expensive tests, procedures, and additional medications, and significantly impacts quality of life at a young age for those with FH. Profoundly, many talked about the impact the death of parent or child in their 40s or 50s, or younger, had on the family.

“You treat this, you get this under control probably saved my life or I could have not [had] as many problems as the rest of my family did. Which ends up costing everybody else more money for your life.” (Participant 10)

Participants’ experiences illustrated the cost of FH at both the individual and societal levels and the value of interventions to improve early diagnosis and effective treatment, given the prevalence of FH, the high risk for ASCVD in this population, and the fact effective treatments are available.

### 3.6 Awareness

Participants felt that it was important to improve awareness of FH so that their lived experiences of delayed diagnosis and undertreatment do not persist. However, participants felt that awareness should not be limited to clinician education through continuing education and training, but also should be focused on targeting individuals at risk for FH and the general public. Often participants reported substantial family history of high cholesterol and ASCVD, but clinicians were unaware that this might be FH. Participants expressed that knowing and understanding the symptoms of FH would have helped them understand why some treatments and lifestyle changes did not work for them and would have expedited the time taken to receive evidence-based care. This was more evident in certain cases; for example, women stated their ASCVD was missed and some participants expressed their clinicians had reservations about screening and treating their children.

“A lot of peoples' stories that we've heard are equivalent to mine where I went to my cardiologist who has been in the field for, I don't know, 50 years. And [my cardiologist] didn't…my lipid panel was through the roof and my LDL was very, very high and [my cardiologist] never said anything about FH or familial hypercholesterolemia.” (Participant 34)

### 3.7 Research & registries

Participants discussed that they want to contribute to research for FH and that research will help to provide answers for the future. Others described the importance of conducting research to improve identification and treatment for individuals with FH. Participants wanted to know about new medications that are on the horizon and expressed the need for medicines that have fewer side effects and better fit patients’ lifestyles. They stated that they would like evidence-based information. One participant discussed how registries will help compare their own specific situation to others. Most participants were able to demonstrate understanding about the genetic nature of FH by describing how the risks affect themselves or their family.

“So just it would be good to know what people are trying. What's going on out there. What is there is there something new on horizon. Just information period.” (Participant 23)

### 3.8 Severe & homozygous FH

A few participants were affected by the rare form of FH called homozygous FH or had severe FH. These participants stressed the need for early identification and that sometimes even when traditional signs are present, like xanthomas, an FH diagnosis can be missed. Participants discussed the difficulties in finding specialists, navigating insurance, and the need for additional treatments to sufficiently lower their cholesterol.

“[My daughter] turns 8 in just a couple of days. If we still weren't getting apheresis, I don't, it wouldn't surprise me if she would have already had a heart attack because her valve is already hardened, with all the medication she is on and everything, it's still doing that, and her, they aged her heart at a 50-year-olds. So, that's with treatment, which obviously I know for her, you know, it's going to be a slower situation. But it's still the situation. The outcome she's having, even though it's a lot faster, that's still the outcome for everyone else even if it takes an extra 10 or 15 years, that's still going to be where you're at.” (Participant 60)

## 4 Discussion

A clear take-away from examining the alignment of lived experiences with FH with the Global Call to Action is that individuals and families who understand their FH diagnosis know what actions are needed to improve diagnosis and care, and where the health system has failed them. The Global Call to Action originated with the convening of over 40 FH advocacy organizations, individuals with FH, and scientific experts from around the world to examine the progress, or lack thereof, towards addressing the priorities outlined in 1998 by the World Health Organization[4]. The lived experiences documented here affirm the priorities set forth by the Global Call to Action. These lived experiences suggest multiple issues that should be addressed to help citizens and those newly diagnosed with FH understand the importance of the condition. It is important to recognize that this work highlights lived experiences from individuals and families with FH who are mostly white, older, and well-educated. The experiences of those with FH from lower socioeconomic status, less education, and minority populations are likely worse.

Family-based care was one of two most often mentioned recommendations that would require health system adjustments but has potential to be a model of greater efficiency for the delivery of clinical care. Family-based care is already being explored for other genetic conditions[9]. Concerns about the health and safety of family members are common and can often motivate individuals to pursue care that could benefit not just themselves, but generations of family[[10], [11], [12], [13]]. Family members also provide support including accompanying patients to appointments, sharing information about FH through the family network, facilitating adherence to evidence-based regimens, and providing emotional support. One implication of this work is that those caring for FH patients, such as health care systems and insurers, should consider implementing and paying for alternative models of care to allow families to undergo care together, as our work suggests families often learn about FH from each other. An example of this approach has shown that when unaffected or affected family members accompany an individual with heart failure to medical visits, self-care improves[14].

Screening, testing, and diagnosis was the other most frequently mentioned recommendation. As FH is inherited, this recommendation and family-based care are closely aligned in the minds of patients. The need to diagnose FH as early as possible and missed opportunities for diagnosis were common themes. Our study and others found genetic testing to be helpful as it provided a diagnosis, but also created strain when family members were reluctant to be tested or there were concerns about life insurance discrimination[15]. More work needs to be done to implement screening programs for FH that lead to earlier diagnosis, as well as research into any potential for genetic discrimination when genetic testing is used at early ages for FH diagnosis.

Additional themes included the need for more treatment options, frustration regarding lack of awareness of FH at every level, and issues related to cost of care and difficulties navigating the health care and insurance systems. These themes are not new to the field of FH and have been cited in previous work[7,[16], [17], [18]].

Clinicians should be aware of the concerns, attitudes, and preferences discussed in these interviews and focus groups, as understanding the experience of living with FH could improve FH awareness and early diagnosis, communication with patients and their families, adherence to medication, and other aspects of FH care. Important elements of the care encounter to be covered include clear and consistent messaging around diagnosis and treatment, understanding of family issues related to FH, and addressing barriers to care. In particular, clinicians and their care extenders should seek to understand and appreciate the family system's history of living with FH and their need for family-based care to provide tailored support and recommendations to meet the family's needs.

Health systems could further improve FH care by supporting alternative care models such as family-based care [19, 20] and programs to help patients achieve cholesterol lowering goals. Payers and government agencies regulating health care should consider reimbursement and regulatory strategies aligned with patient concerns and preferences in order to address the cost of care and the value of early diagnosis and treatment of FH for ASCVD prevention. Additionally, health systems and policy makers need to explore care models that protect patient privacy while reducing barriers for sharing clinically actionable information among clinicians treating members of a family with FH. Developing pathways for communication within a healthcare system to alert clinicians caring for relatives of an individual diagnosed with FH can reduce the burden of disclosure on patients, the complexities at-risk relatives face when trying to follow up about their FH-related health risks, and improve care coordination that support family-based care.

Finally, both the Global Call to Action and these lived experiences highlight the importance of addressing FH as a public health priority and funding the research and interventions necessary to make meaningful progress towards preventing ASCVD and premature death caused by FH.

## 5 Public health implications

The FH Global Call to Action was driven by the persistent unmet needs of those living with FH in receiving a timely diagnosis, appropriate care and management of this preventable disease, and support to prevent early morbidity and mortality. Patient- and family-centric perspectives underline the need to prioritize FH as a global public health concern, support FH education and advocacy efforts, fund FH research, and innovate solutions for screening, testing, diagnosis, and family-based care.

### Funding

Research reported in this publication was supported by the 10.13039/100000050National Heart, Lung, and Blood Institute of the National Institutes of Health under award number R01HL148246 and K12HL137942. The findings and conclusions in this paper are those of the authors and do not necessarily represent the official positions of the National Institutes of Health.

## Contributions

Laney K. Jones, PharmD designed the study, conducted the analysis, wrote the paper and provided final review. Nicole Walters helped with the analysis, reviewed and provided final review of the paper. Andrew Brangan helped with the analysis, reviewed and provided final review of the paper. Catherine D. Ahmed, MBA helped with the analysis, reviewed and provided final review of the paper.  Katherine A. Wilemon, BS helped with the analysis, reviewed and provided final review of the paper. Gemme Campbell-Salome, PhD helped with the analysis, reviewed and provided final review of the paper. Alanna K. Rahm, PhD helped with the analysis, reviewed and provided final review of the paper. Samuel S. Gidding, MD helped with the design and analysis, reviewed and provided final review of the paper.  Amy C. Sturm, MS helped with the design and analysis, reviewed and provided final review of the paper.

## Conflicts of Interest

The authors had access to all the study data, take responsibility for the accuracy of the analysis, and had authority over manuscript preparation and the decision to submit the manuscript for publication. None of the authors have any competing interests to disclose.
